# Materialism and Subjective Well-Being Among Chinese Higher Vocational College Students: The Mediating Role of Basic Psychological Need Satisfaction and the Moderating Role of Self-Compassion

**DOI:** 10.3390/ejihpe16070099

**Published:** 2026-07-12

**Authors:** Zhengqi Wei, Hualing Miao, Cheng Guo

**Affiliations:** 1 Faculty of Psychology, Southwest University, Chongqing 400715, China; ellawei@email.swu.edu.cn; 2 Office of Academic Affairs and Faculty of Teacher Education, West Yunnan University, Lincang 677000, China; 3 Department of Psychology, Honghe University, Mengzi 661199, China

**Keywords:** subjective well-being, materialism, basic psychological needs, self-compassion, higher vocational college students

## Abstract

Subjective well-being is a crucial indicator of higher vocational college students’ mental health, while materialistic values may be associated with poorer well-being. This study examined the mechanisms through which materialism is related to subjective well-being among Chinese higher vocational college students, with subjective well-being operationalized using life satisfaction and depressive symptoms, focusing on the mediating role of basic psychological need satisfaction (BPNS) and the moderating role of self-compassion. The final analytic sample included 4012 higher vocational college students from southwestern China (*M*_age_ = 18.79 years, *SD* = 0.96, 37.1% male), and the data were analyzed using structural equation modeling with robust maximum likelihood estimation and latent moderated structural equations. Results indicated that materialism was associated with lower life satisfaction and higher depressive symptoms. BPNS partially mediated the associations between materialism and both outcomes. Self-compassion moderated the associations between BPNS and both life satisfaction and depressive symptoms, but the moderated mediation effect was significant only for depressive symptoms. Specifically, self-compassion weakened the indirect association between materialism and depressive symptoms through BPNS. These findings contribute to SDT-based research by integrating vulnerability and resilience perspectives and suggest that self-compassion may be particularly relevant to lower depressive symptoms associated with lower BPNS among higher vocational college students.

## 1. Introduction

Mental health and well-being among young people have become major public health concerns worldwide (World Health Organization, 2025). Emerging adulthood, particularly the college years, represents a critical developmental stage characterized by identity exploration, increasing independence, and the establishment of long-term goals (Arnett, 2000). During this period, individuals experience substantial academic, interpersonal, and social pressures, making them especially vulnerable to psychological difficulties such as depression, anxiety, and reduced life satisfaction (Beiter et al., 2015; Stallman, 2010). This issue may be particularly relevant for higher vocational college students, who are often preparing for the school-to-work transition and may face salient concerns about career development and employability (H. Li et al., 2024; X.-H. Wang et al., 2023). Consequently, identifying factors associated with higher vocational college students’ well-being has become an important issue in psychological research.

Subjective well-being refers to individuals’ overall evaluations of their lives and includes both cognitive and affective components. Cognitive well-being is typically reflected in life satisfaction, whereas affective well-being refers to the experience of positive and negative emotions (Diener, 1984; Sagiv & Schwartz, 2000). In empirical research on subjective well-being, depressive symptoms have often been used as negative indicators of well-being (Dejonckheere et al., 2022; Nartova-Bochaver et al., 2019) because they are closely related to negative affective experiences, such as depressed mood, anhedonia, and emotional distress (Kroenke et al., 2001). Previous research on materialism has often adopted a broad approach to well-being by including both positive indicators, such as life satisfaction, and negative indicators, such as depression or depressive symptoms (Dittmar et al., 2014). Drawing on this broader operationalization, the present study examined subjective well-being using two complementary indicators: life satisfaction and depressive symptoms. Life satisfaction served as the cognitive and positive indicator of subjective well-being, whereas depressive symptoms served as a negative indicator of subjective well-being, with higher depressive symptoms reflecting poorer subjective well-being. Although both indicators were used to operationalize subjective well-being, life satisfaction reflects cognitive evaluations of one’s life, whereas depressive symptoms reflect negative affective, cognitive and somatic experiences; therefore, they were examined as separate outcomes. This approach allows for a more nuanced understanding of whether the proposed mechanisms operate similarly or differently across positive and negative indicators of subjective well-being.

One factor that may be particularly relevant to young people’s well-being is materialism. With rapid economic development and the expansion of consumer culture, individuals are increasingly exposed to social messages emphasizing money, possessions, status, and appearance (Cleveland et al., 2023). Globalization and social media have further intensified exposure to materialistic values, particularly among younger generations (Cleveland et al., 2022; Ho et al., 2019).

Materialism has often been conceptualized as a value orientation consisting of three dimensions: centrality, happiness, and success (Richins & Dawson, 1992). Individuals high in materialism tend to regard possessions as central life goals and view material acquisition as an important pathway to happiness and personal success. However, empirical findings consistently indicate that materialistic pursuits often fail to enhance well-being. Research has shown that materialism is associated not only with lower life satisfaction and happiness, but also with depression, loneliness, anxiety, and low self-esteem (Dittmar et al., 2014; Mueller et al., 2011; Pieters, 2013). This paradoxical discrepancy between the expected benefits of material success and its actual psychological consequences highlights the need to better understand the mechanisms through which materialism is associated with life satisfaction and depressive symptoms.

From the perspective of Self-Determination Theory (SDT; Deci & Ryan, 2000), well-being depends largely on the satisfaction of three basic psychological needs: autonomy, competence, and relatedness. Autonomy refers to experiencing one’s behavior as self-endorsed and volitional; competence refers to feeling capable and effective; and relatedness refers to feeling connected to others (Deci & Ryan, 2014). SDT further distinguishes between intrinsic goals and extrinsic goals. Materialistic values emphasize extrinsic aspirations such as wealth, status, and image, which may interfere with the fulfillment of individuals’ basic psychological needs. In contrast, intrinsic aspirations focus on personal growth, close relationships, and community contribution, all of which are more likely to satisfy the needs for autonomy, competence, and relatedness (Kasser & Ryan, 1996). Individuals who prioritize material success may become overly focused on external approval and social comparison, thereby neglecting intrinsically meaningful goals and relationships. Such a value orientation may reduce opportunities for authentic self-expression, meaningful interpersonal connection, and experiences of mastery, ultimately being associated with lower basic psychological need satisfaction (BPNS; Chen et al., 2015; Vansteenkiste et al., 2006). Accordingly, BPNS may represent an important mediating mechanism linking materialism to lower life satisfaction and higher depressive symptoms (Chen et al., 2015; Niemiec et al., 2009; R. Wang et al., 2017).

Although previous studies have demonstrated that BPNS mediates the relationship between materialism and well-being, most existing research has primarily focused on identifying why materialism is associated with poorer subjective well-being, while paying comparatively little attention to why some highly materialistic individuals nevertheless maintain relatively good psychological functioning despite experiencing lower need satisfaction (Dittmar et al., 2014; Kasser, 2016). This limitation suggests that current knowledge of the materialism–well-being relationship remains largely deficit-oriented, emphasizing risk factors while overlooking potential resilience processes (Masten, 2001). From a resilience perspective, the psychological consequences of low need satisfaction may not be uniform across individuals (Masten, 2001). Some individuals appear capable of maintaining relatively favorable subjective well-being even when their basic psychological needs are not fully fulfilled. The same level of unmet psychological needs may be associated with lower life satisfaction or more severe depressive symptoms for some students but weaker negative associations for others, depending on their personal psychological resources. Identifying personal resources that may attenuate the negative associations of unmet psychological needs may therefore provide a more comprehensive understanding of the conditions under which materialism is linked to poorer subjective well-being.

One potential psychological resource that may serve this protective function is self-compassion. It involves responding to personal difficulties with warmth and understanding, recognizing such experiences as part of the common human condition, and maintaining a balanced awareness of negative emotions rather than becoming overwhelmed by them (Neff, 2003a). A growing body of research indicates that individuals with higher levels of self-compassion tend to report fewer symptoms of depression and anxiety, along with greater life satisfaction and overall psychological well-being (Barnard & Curry, 2011; Neff et al., 2007; Zessin et al., 2015).

The potential protective role of self-compassion may also be understood from the perspective of SDT. Psychological need satisfaction is generally regarded as an essential source of well-being (Ryan & Deci, 2017). However, individuals may differ in the extent to which unmet needs impair their psychological functioning. Self-compassion may serve as an important psychological resource in this process (Neff, 2003a; Neff et al., 2007). Individuals with high levels of self-compassion tend to respond to personal difficulties with kindness, acceptance, and emotional balance rather than self-criticism and rumination (Neff, 2003a). Consequently, even when their psychological needs are not fully satisfied, they may be better able to regulate negative emotions and maintain positive psychological functioning (Inwood & Ferrari, 2018; Terry & Leary, 2011). In contrast, individuals with low self-compassion may be more vulnerable when their psychological needs are unmet because they are more likely to engage in harsh self-evaluation and emotional over-identification (Barnard & Curry, 2011).

The moderating role of self-compassion may be particularly relevant to the pathways from BPNS to the two indicators of subjective well-being. BPNS reflects the extent to which individuals experience autonomy, competence, and relatedness in their daily lives (Chen et al., 2015; Ryan & Deci, 2000), whereas self-compassion reflects how individuals respond to difficulties, shortcomings, and distressing experiences (Neff, 2003a). Thus, self-compassion may not necessarily alter the extent to which materialistic values are associated with lower BPNS; rather, it may shape how students emotionally respond when their basic psychological needs are not fully satisfied. Rather than viewing individuals as passive recipients of the negative consequences associated with materialistic values, a self-compassion perspective highlights the possibility that people possess psychological resources that enable them to cope more effectively with unmet needs and emotional distress (Inwood & Ferrari, 2018; Neff, 2003a; Terry & Leary, 2011). In this regard, self-compassion may function as a resilience-related factor that attenuates the negative psychological associations linked to materialistic value orientations. At the same time, it is important to acknowledge that self-compassion may also be conceptualized in other ways, such as a mediator, antecedent variable, covariate, or parallel psychological resource. The present study focused on its moderating role based on resilience and emotion-regulation perspectives, while recognizing that alternative models should be examined in future longitudinal research.

In addition, the present study focuses on Chinese higher vocational college students for both theoretical and practical reasons. China has experienced rapid economic growth and increasing consumerism over the past several decades, creating a social environment in which materialistic values may become particularly salient among young adults (Durvasula & Lysonski, 2010; Podoshen et al., 2011; C. L. Wang & Lin, 2009). Higher vocational colleges constitute an important sector of Chinese higher education and enroll many young adults who are preparing for employment and social transition (H. Li et al., 2024; X.-H. Wang et al., 2023). Compared with students in more research-oriented universities, higher vocational college students may face distinctive contextual pressures related to employability, social mobility, and future career prospects. These concerns may make material success, social status, and achievement especially salient for this group (H. Li et al., 2024; X.-H. Wang et al., 2023; Zhai et al., 2021). Furthermore, cultural factors may influence both the expression of materialism and the psychological functions of self-compassion (Ger & Belk, 1996; Neff et al., 2008). Examining these relationships within the Chinese higher vocational education context may therefore contribute to a broader understanding of the mechanisms linking materialism and subjective well-being. Moreover, understanding how materialistic values, BPNS, and self-compassion are associated with higher vocational college students’ life satisfaction and depressive symptoms may inform mental health promotion and need-supportive educational practices in Chinese higher education.

### The Current Study

Drawing upon Self-Determination Theory, the present study aims not only to examine the negative indirect associations linking materialism to life satisfaction and depressive symptoms through BPNS, but also to identify a potential resilience-related mechanism through which these associations may be attenuated. By integrating both risk and protective perspectives within a single framework, the present study extends previous SDT-based research on materialism beyond the question of why materialism is associated with poorer subjective well-being to address when and for whom these negative associations may be attenuated. Furthermore, by simultaneously examining life satisfaction and depressive symptoms, the present study distinguishes between positive cognitive and negative symptom-related indicators of subjective well-being, allowing for a more nuanced understanding of the protective role of self-compassion. Within this theoretically grounded framework, and recognizing the cross-sectional nature of the data, the present study tested the following hypotheses regarding indirect and conditional associations:

**Hypothesis** **1a.** *Materialism will be negatively associated with life satisfaction.*

**Hypothesis** **1b.** *Materialism will be positively associated with depressive symptoms.*

**Hypothesis** **2a.** *Basic psychological need satisfaction will mediate the association between materialism and life satisfaction.*

**Hypothesis** **2b.** *Basic psychological need satisfaction will mediate the association between materialism and depressive symptoms.*

**Hypothesis** **3a.** *Self-compassion will moderate the association between BPNS and life satisfaction.*

**Hypothesis** **3b.** *Self-compassion will moderate the association between BPNS and depressive symptoms.*

In addition, we further explored whether the indirect associations from materialism to life satisfaction and depressive symptoms through BPNS varied across levels of self-compassion.

## 2. Materials and Methods

### 2.1. Participants and Procedure

Data were collected from six higher vocational colleges in southwestern China. A total of 4706 students completed the questionnaire. After sequential data-quality screening, 694 responses were excluded because of extremely rapid responding (average response time ≤ 2 s per item; *n* = 444), duplicate submissions (*n* = 41), or clear patterned responding, such as highly repetitive, invariant, or regular response patterns (*n* = 209). The final analytic sample consisted of 4012 valid participants, yielding a valid response rate of 85.25%. The participants ranged in age from 16 to 25 years, with an average age of 18.79 years (*SD* = 0.96). Concretely, 95.3% of the participants were 20 years old or younger (*n* = 3825). Of the participants, 65.5% were in grade 1 (*n* = 2629), 29.0% were in grade 2 (*n* = 1164), and 5.5% were in grade 3 (*n* = 219). Of the participants, 37.1% were male (*n* = 1488) and 62.9% were female (*n* = 2524). Regarding family structure, 21.6% were only children (*n* = 868), while 78.4% had siblings (*n* = 3144). In terms of residential background, 33.2% lived in urban areas (*n* = 1332), and 66.8% resided in rural regions (*n* = 2680). Regarding subjective socioeconomic status (SES), 21.2% of participants were facing difficulties (*n* = 849), 36.8% of participants were below average (*n* = 1476), 41.3% of participants were average (*n* = 1656), while 0.8% of participants were very comfortable (*n* = 31).

This study was approved by the Ethics Committee of the Faculty of Psychology at Southwest University of China (IRB protocol number: SWU-ECHR-20250241). Permission was obtained from the participating schools, and informed consent was obtained from all participants and from the parents or legal guardians of participants under 18 years of age before data collection. Participants were assured of the confidentiality of their personal information and responses. Furthermore, every effort was made to ensure that no physical or psychological harm would be caused to the participants.

### 2.2. Measures

#### 2.2.1. Materialism

Materialism was assessed using the 13-item Material Values Scale (MVS), originally developed by Richins and Dawson (1992) and later revised by J. Li and Guo (2009). The scale measures three dimensions of materialism: success, centrality, and happiness. Participants responded to each item on a 5-point Likert-type scale ranging from 1 (“strongly disagree”) to 5 (“strongly agree”). After reverse scoring Items 2, 4, 5, 6, and 10, all 13 items were used to compute the materialism score, with higher scores indicating stronger materialistic values.

A confirmatory factor analysis (CFA) was conducted to evaluate the measurement structure of the scale. The model specified the three theoretical factors of materialism and included a method factor for the reverse-worded items. The model showed acceptable fit to the data, χ^2^(51) = 567.677, CFI = 0.941, TLI = 0.910, RMSEA = 0.050, 90% CI [0.047, 0.054], SRMR = 0.035. In the SEM analyses, the three dimension scores—success, centrality, and happiness—were used as observed indicators of the latent materialism construct. The scale demonstrated acceptable internal consistency in the present sample, with Cronbach’s α = 0.754.

#### 2.2.2. Basic Psychological Need Satisfaction

Basic psychological need satisfaction (BPNS) was measured using the need satisfaction items from the Basic Psychological Need Satisfaction and Frustration Scale (BPNSFS) developed by Chen et al. (2015). The scale contains 12 items assessing satisfaction of the needs for competence, autonomy, and relatedness. Participants rated each item on a 5-point Likert-type scale ranging from 1 (“completely untrue”) to 5 (“completely true”). Higher mean scores indicate greater BPNS.

A three-factor CFA model corresponding to competence, autonomy, and relatedness showed acceptable fit to the data, χ^2^(51) = 1150.639, CFI = 0.957, TLI = 0.944, RMSEA = 0.073, 90% CI [0.070, 0.077], SRMR = 0.031. In the SEM analyses, the three dimension scores—competence, autonomy, and relatedness—were used as observed indicators of the latent BPNS construct. The scale showed good internal consistency in the present sample, with Cronbach’s α = 0.918.

#### 2.2.3. Self-Compassion

Self-compassion was measured using the Chinese version of the Self-Compassion Scale (SCS), originally developed by Neff (2003b) and later adapted into Chinese by Gong et al. (2014). The 12-item version used in the present study assesses three components of self-compassion: self-kindness, common humanity, and mindfulness. Participants responded to each item on a 5-point Likert-type scale ranging from 1 (“never”) to 5 (“always”). Items 2, 4, 5, 8, and 11 were reverse-scored, with higher mean scores indicating greater self-compassion.

A three-factor CFA model corresponding to self-kindness, common humanity, and mindfulness showed acceptable fit to the data, χ^2^(47) = 1208.140, CFI = 0.943, TLI = 0.919, RMSEA = 0.078, 90% CI [0.075, 0.082], SRMR = 0.062. In the SEM analyses, the three dimension scores—self-kindness, common humanity, and mindfulness—were used as observed indicators of the latent self-compassion construct. The scale demonstrated acceptable internal consistency in the present sample, with Cronbach’s α = 0.799.

#### 2.2.4. Life Satisfaction

Life satisfaction was assessed using a three-item version adapted from the Satisfaction with Life Scale (SWLS), originally developed by Diener et al. (1985). The Chinese version of the SWLS was adapted by Xiong and Xu (2009). In the present survey, three items were administered to assess participants’ global cognitive evaluation of their lives, including perceived life conditions, overall life satisfaction, and the attainment of important life goals. A sample item is “I am satisfied with my life.” Participants responded to each item on a 5-point Likert-type scale ranging from 1 (“completely untrue”) to 5 (“completely true”). Consistent with the conceptualization in the Introduction, life satisfaction was treated as the cognitive and positive indicator of subjective well-being. Higher mean scores indicate greater life satisfaction.

Given that the three-item one-factor model was just-identified, global CFA fit indices were not interpreted. All standardized factor loadings were statistically significant and ranged from 0.660 to 0.833. In the SEM analyses, the three items were used as observed indicators of the latent life satisfaction construct. The scale demonstrated acceptable internal consistency in the present sample, with Cronbach’s α = 0.775.

To further evaluate the adequacy of the three-item measure, an additional validation analysis was conducted in a separate sample of 1627 participants who completed the full five-item SWLS. The three-item version showed acceptable internal consistency (Cronbach’s α = 0.799), and the full five-item version showed good internal consistency (Cronbach’s α = 0.862). The three-item score was strongly correlated with the full five-item score (*r* = 0.947, *p* < 0.001) and was also substantially correlated with the mean score of the two remaining SWLS items (*r* = 0.719, *p* < 0.001), supporting its use as a brief indicator of life satisfaction.

#### 2.2.5. Depressive Symptoms

Depressive symptoms were measured using the Patient Health Questionnaire-9 (PHQ-9; Kroenke et al., 2001). The PHQ-9 contains nine items assessing the frequency of depressive symptoms over the past two weeks. Each item is rated on a 4-point scale ranging from 0 (“not at all”) to 3 (“nearly every day”). Higher mean scores indicate more severe depressive symptoms. Consistent with the conceptualization in the Introduction, depressive symptoms were used as a negative indicator of subjective well-being in the present non-clinical higher vocational college student sample, as they are closely related to negative affective experiences such as depressed mood, anhedonia, and emotional distress, while also reflecting broader cognitive and somatic symptoms. A one-factor CFA model for the PHQ-9 showed acceptable fit to the data, χ^2^(26) = 674.394, CFI = 0.958, TLI = 0.942, RMSEA = 0.079, 90% CI [0.074, 0.084], SRMR = 0.031. The scale demonstrated good internal consistency in the present sample, with Cronbach’s α = 0.887.

#### 2.2.6. Demographic Variables

Participants reported demographic information including age, gender, grade, residential background, only-child status, and subjective socioeconomic status. Gender was coded as 0 = male and 1 = female. Residential background was coded as 0 = urban and 1 = rural, and only-child status was coded as 0 = non-only child and 1 = only child. Subjective socioeconomic status was assessed with one item asking participants to report their perceived family economic condition, coded as 0 = facing difficulties, 1 = below average, 2 = average, and 3 = very comfortable, with higher scores indicating higher perceived family economic status.

### 2.3. Data Analysis

Data analyses were conducted using IBM SPSS 25.0 and Mplus 8.3. Descriptive statistics, reliability coefficients, collinearity diagnostics, common method bias tests, and Pearson correlation analyses were first conducted as preliminary analyses. Confirmatory factor analyses (CFAs) were then performed to evaluate the measurement structure of each scale and the overall five-factor measurement model. Cronbach’s α was used to assess internal consistency.

Structural equation modeling (SEM) with robust maximum likelihood estimation (MLR) was used to test the mediation and moderated mediation models. Materialism, BPNS, self-compassion, life satisfaction, and depressive symptoms were modeled as latent variables. Materialism, BPNS, and self-compassion were indicated by their respective dimension scores; life satisfaction was indicated by its three items; and depressive symptoms were indicated by three item parcels. Following an alternating-item parceling approach, the depressive symptom parcels were formed by averaging Items 1, 4, and 7; Items 2, 5, and 8; and Items 3, 6, and 9 of the PHQ-9, respectively.

Based on theoretical relevance and preliminary correlation analyses, age, subjective socioeconomic status, residential background, and only-child status were included as covariates in the main structural models. Gender was not included because it was not significantly associated with the focal outcome variables in the preliminary analyses. Grade was also not included because it largely overlapped with age as an indicator of students’ developmental and educational stage; therefore, age was retained as the more fine-grained continuous indicator to maintain model parsimony.

The mediation models were first estimated without latent interaction terms to examine the indirect associations from materialism to life satisfaction and depressive symptoms through BPNS. The moderated mediation models were then tested using the latent moderated structural equations (LMS) approach in Mplus (Klein & Moosbrugger, 2000; Maslowsky et al., 2015). Specifically, the latent interaction between BPNS and self-compassion was estimated to examine whether self-compassion moderated the associations between BPNS and the two well-being outcomes. Because LMS models with latent interactions do not provide conventional global fit indices, baseline models without latent interaction terms were compared with the corresponding latent interaction models using H0 loglikelihood values, AIC, BIC, and MLR-adjusted likelihood ratio tests.

Indirect effects, conditional indirect effects, simple slopes, and indices of moderated mediation were calculated using model constraints in Mplus. Low and high levels of self-compassion were defined as one standard deviation below and above the latent mean, respectively. Unless otherwise specified, reported path coefficients are unstandardized. Statistical significance was evaluated at *p* < 0.05.

## 3. Results

### 3.1. Collinearity Diagnostics and Common Method Bias Analyses

Collinearity diagnostics indicated that the core predictor variables exhibited tolerance values ranging from 0.712 to 0.887, each exceeding the recommended threshold of 0.2, while variance inflation factors varied between 1.127 and 1.405, all below the commonly used critical value of 10 (Hair et al., 2019; O’Brien, 2007). These results suggest that multicollinearity was unlikely to be a concern among the predictors.

We also considered the possibility of common method bias, as all psychological variables were measured through self-report. To address this concern, we performed Harman’s (1976) single-factor test. Exploratory factor analysis was conducted using unrotated principal components based on all scale items included in the study. The results revealed nine factors with eigenvalues greater than 1, with the first factor accounting for 22.733% of the variance, which is well below the 50% criterion (Podsakoff & Organ, 1986). Additionally, the single-factor confirmatory factor analysis demonstrated poor model fit: χ^2^(1127) = 36,280.832, CFI = 0.466, TLI = 0.443, RMSEA = 0.088, SRMR = 0.103. Taken together, these results suggest that common method bias was unlikely to fully account for the observed associations.

### 3.2. Descriptive Statistics and Correlations

The descriptive statistics and correlations for the studied variables are presented in Table 1. Materialism was significantly and negatively associated with BPNS, self-compassion, and life satisfaction, and was significantly and positively associated with depressive symptoms. BPNS was positively associated with life satisfaction and self-compassion, and negatively associated with depressive symptoms. Life satisfaction was negatively associated with depressive symptoms and positively associated with self-compassion.

With regard to covariates, age was significantly associated with materialism, BPNS, depressive symptoms, and self-compassion, but not with life satisfaction. Gender was significantly associated with materialism and self-compassion, but was not significantly associated with BPNS, life satisfaction, or depressive symptoms. Subjective SES was significantly associated with BPNS, depressive symptoms, life satisfaction, and self-compassion, but not with materialism. Residence was significantly associated with materialism, BPNS, life satisfaction, and self-compassion, whereas its association with depressive symptoms was not significant. Only-child status was significantly associated with BPNS, life satisfaction, and self-compassion, but not with materialism or depressive symptoms. Based on theoretical considerations and these preliminary correlation results, age, subjective SES, residence, and only-child status were included as covariates in the subsequent structural equation models.

### 3.3. Measurement Model

Before testing the structural models, we conducted a confirmatory factor analysis to evaluate the five-factor measurement model. The measurement model included materialism, BPNS, self-compassion, life satisfaction, and depressive symptoms as latent constructs. Specifically, materialism was indicated by the dimension scores for success, centrality, and happiness; BPNS was indicated by competence, autonomy, and relatedness; self-compassion was indicated by self-kindness, common humanity, and mindfulness; life satisfaction was indicated by its three items; and depressive symptoms were indicated by three item parcels.

Based on modification indices and theoretical plausibility, the residuals of the mindfulness and common humanity indicators within the self-compassion construct were allowed to correlate, and the same residual covariance was retained in subsequent structural models. This adjustment was limited to indicators from the same latent construct and did not involve any cross-construct loadings, thereby preserving the theoretical distinctiveness of the latent variables.

The five-factor measurement model demonstrated acceptable fit: χ^2^(79) = 842.461, CFI = 0.964, TLI = 0.952, RMSEA = 0.049, 90% CI [0.046, 0.052], SRMR = 0.042. All standardized factor loadings were statistically significant. Specifically, the standardized factor loadings ranged from 0.565 to 0.873 for materialism, 0.778 to 0.897 for BPNS, 0.526 to 0.773 for self-compassion, 0.629 to 0.867 for life satisfaction, and 0.839 to 0.881 for depressive symptoms. These results suggested that the hypothesized five-factor structure was generally supported and suitable for subsequent structural analyses.

### 3.4. Mediation and Moderated Mediation Analyses

Mediation and moderated mediation analyses were conducted following the analytic strategy described above. All models controlled for age, subjective SES, residence, and only-child status. The results of the mediation models are reported first, followed by the LMS-based moderated mediation models.

#### 3.4.1. Mediation Models

**Figure 1:**
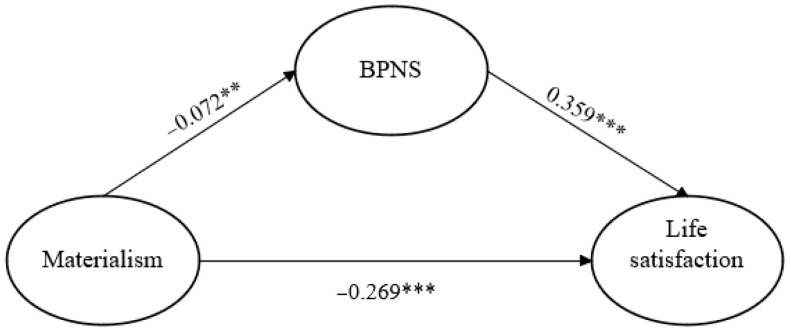
Mediation model of BPNS in the association between materialism and life satisfaction. Note. BPNS = basic psychological need satisfaction. Values represent unstandardized path coefficients. ** *p* < 0.01. *** *p* < 0.001.

For life satisfaction, the mediation model demonstrated acceptable fit: χ^2^(87) = 1227.916, CFI = 0.929, TLI = 0.907, RMSEA = 0.057, SRMR = 0.047. Materialism was negatively associated with BPNS, *b* = −0.072, *SE* = 0.024, *p* = 0.002. BPNS was positively associated with life satisfaction, *b* = 0.359, *SE* = 0.028, *p* < 0.001. The direct association between materialism and life satisfaction remained significant, *b* = −0.269, *SE* = 0.026, *p* < 0.001. The indirect association from materialism to life satisfaction through BPNS was significant, *b* = −0.026, *SE* = 0.009, *p* = 0.003, indicating partial mediation. The mediation model for life satisfaction is presented in Figure 1.

**Figure 2:**
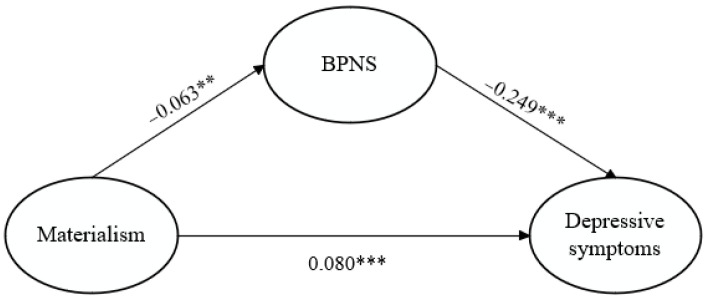
Mediation model of BPNS in the association between materialism and depressive symptoms. Note. BPNS = basic psychological need satisfaction. Values represent unstandardized path coefficients. ** *p* < 0.01. *** *p* < 0.001.

For depressive symptoms, the mediation model demonstrated good fit: χ^2^(87) = 925.503, CFI = 0.955, TLI = 0.941, RMSEA = 0.049, SRMR = 0.045. Materialism was negatively associated with BPNS, *b* = −0.063, *SE* = 0.024, *p* = 0.008. BPNS was negatively associated with depressive symptoms, *b* = −0.249, *SE* = 0.020, *p* < 0.001. The direct association between materialism and depressive symptoms remained significant, *b* = 0.080, *SE* = 0.018, *p* < 0.001. The indirect association from materialism to depressive symptoms through BPNS was also significant, *b* = 0.016, *SE* = 0.006, *p* = 0.009, indicating partial mediation. The mediation model for depressive symptoms is presented in Figure 2.

#### 3.4.2. Moderated Mediation Models

**Figure 3:**
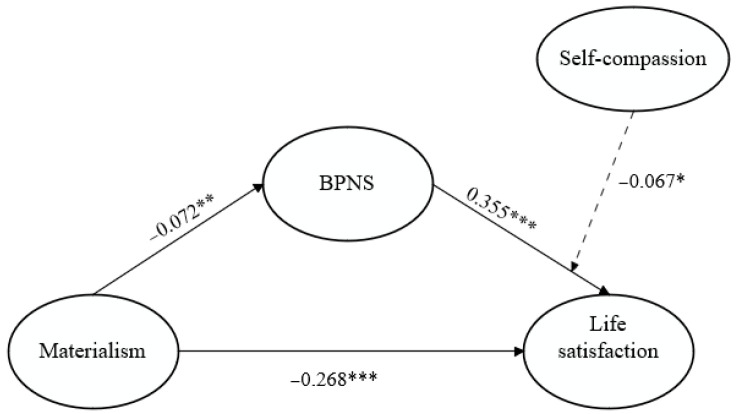
Moderation model for life satisfaction. Note. The dashed arrow indicates the moderating effect of self-compassion on the association between BPNS and life satisfaction. BPNS = basic psychological need satisfaction. Values represent unstandardized path coefficients. Although the BPNS × self-compassion interaction was significant, the index of moderated mediation was not statistically significant. * *p* < 0.05. ** *p* < 0.01. *** *p* < 0.001.

For life satisfaction, adding the BPNS × self-compassion interaction produced limited improvement relative to the mediation model. The H0 loglikelihood increased from −46,314.228 to −46,311.232, and AIC decreased from 92,730.457 to 92,726.463, whereas BIC slightly increased from 93,051.606 to 93,053.909. The MLR-adjusted likelihood ratio test was significant, χ^2^(1) = 4.602, *p* = 0.032. The BPNS × self-compassion interaction significantly predicted life satisfaction, *b* = −0.067, *SE* = 0.031, *p* = 0.031, indicating that self-compassion moderated the association between BPNS and life satisfaction. The moderation model for life satisfaction is presented in Figure 3.

**Figure 4:**
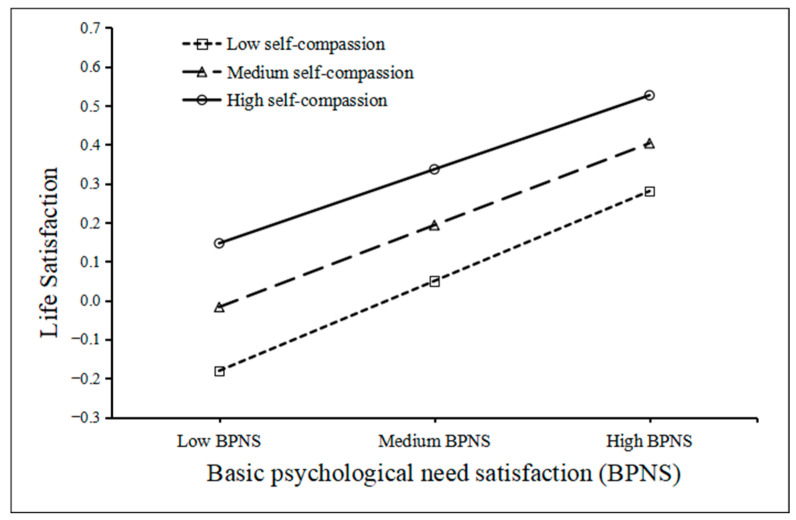
Simple slopes of the association between BPNS and life satisfaction at low, mean, and high levels of self-compassion. Note. BPNS = basic psychological need satisfaction. Low and high self-compassion represent one standard deviation below and above the mean, respectively.

Simple slope analyses showed that BPNS was positively associated with life satisfaction at low, mean, and high levels of self-compassion, although the association became slightly weaker as self-compassion increased. Specifically, the association was strongest at low self-compassion, *b* = 0.390, *SE* = 0.029, *p* < 0.001, followed by mean self-compassion, *b* = 0.355, *SE* = 0.028, *p* < 0.001, and high self-compassion, *b* = 0.321, *SE* = 0.035, *p* < 0.001. The simple slopes are shown in Figure 4.

The conditional indirect association from materialism to life satisfaction through BPNS was significant at low self-compassion, indirect = −0.028, *SE* = 0.009, *p* = 0.003, at mean self-compassion, indirect = −0.025, *SE* = 0.009, *p* = 0.003, and at high self-compassion, indirect = −0.023, *SE* = 0.008, *p* = 0.003. However, the index of moderated mediation was not statistically significant, index = 0.005, *SE* = 0.003, *p* = 0.088. Thus, although self-compassion moderated the BPNS–life satisfaction association, the moderated mediation effect was not supported for life satisfaction.

**Figure 5:**
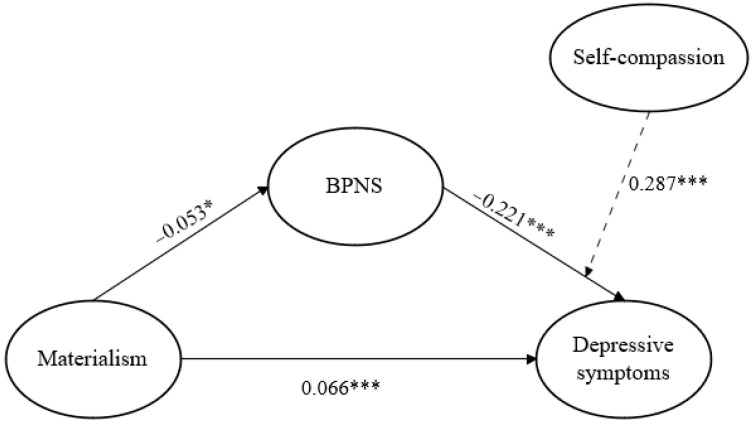
Moderated mediation model for depressive symptoms. Note. The dashed arrow indicates the moderating effect of self-compassion on the association between BPNS and depressive symptoms. BPNS = basic psychological need satisfaction. Values represent unstandardized path coefficients. * *p* < 0.05. *** *p* < 0.001.

For depressive symptoms, adding the BPNS × self-compassion interaction improved the model relative to the mediation model. The H0 loglikelihood increased from −36,257.354 to −36,141.917, and AIC and BIC decreased from 72,616.708 and 72,937.858 to 72,387.834 and 72,715.280, respectively. The MLR-adjusted likelihood ratio test was significant, χ^2^(1) = 145.705, *p* < 0.001. The BPNS × self-compassion interaction significantly predicted depressive symptoms, *b* = 0.287, *SE* = 0.025, *p* < 0.001, indicating that self-compassion moderated the association between BPNS and depressive symptoms. The moderated mediation model for depressive symptoms is presented in Figure 5.

**Figure 6:**
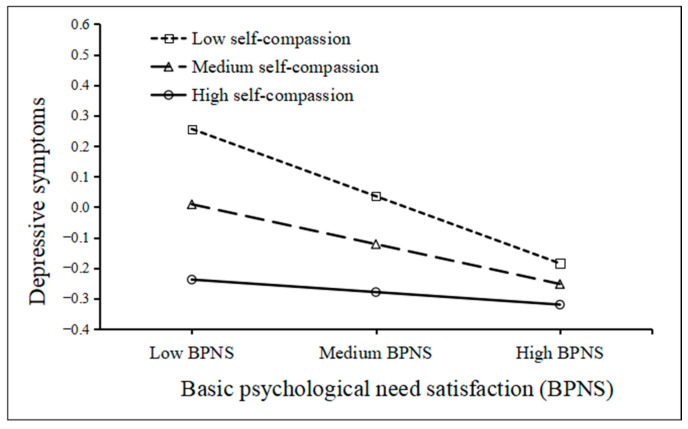
Simple slopes of the association between BPNS and depressive symptoms at low, mean, and high levels of self-compassion. Note. BPNS = basic psychological need satisfaction. Low and high self-compassion represent one standard deviation below and above the mean, respectively.

Simple slope analyses showed that BPNS was negatively associated with depressive symptoms at low, mean, and high levels of self-compassion, but this association became weaker as self-compassion increased. Specifically, the association was strongest at low self-compassion, *b* = −0.373, *SE* = 0.023, *p* < 0.001, weaker at mean self-compassion, *b* = −0.221, *SE* = 0.018, *p* < 0.001, and weakest at high self-compassion, *b* = −0.070, *SE* = 0.021, *p* = 0.001. The simple slopes are shown in Figure 6.

The conditional indirect association from materialism to depressive symptoms through BPNS was significant at low self-compassion, indirect = 0.020, *SE* = 0.009, *p* = 0.026, and at mean self-compassion, indirect = 0.012, *SE* = 0.005, *p* = 0.028, but was not significant at high self-compassion, indirect = 0.004, *SE* = 0.002, *p* = 0.076. The index of moderated mediation was significant, index = −0.015, *SE* = 0.007, *p* = 0.026. These results indicated that self-compassion significantly weakened the indirect association between materialism and depressive symptoms through BPNS.

As a supplementary analysis, we further addressed the possibility that self-compassion may also moderate the first-stage path from materialism to BPNS by testing alternative first-stage moderated mediation models. The results did not support this alternative specification. In the life satisfaction model, the materialism × self-compassion interaction did not significantly predict BPNS, *b* = 0.029, *SE* = 0.036, *p* = 0.424, and the index of moderated mediation was not significant, index = 0.010, *SE* = 0.013, *p* = 0.428. Similarly, in the depressive symptoms model, the materialism × self-compassion interaction did not significantly predict BPNS, *b* = 0.045, *SE* = 0.037, *p* = 0.233, and the index of moderated mediation was not significant, index = −0.011, *SE* = 0.009, *p* = 0.242. These supplementary analyses provided additional support for the focus on the theoretically proposed second-stage moderation model.

Overall, BPNS significantly mediated the associations between materialism and both life satisfaction and depressive symptoms. Self-compassion moderated the associations between BPNS and both indicators of subjective well-being. However, the moderated mediation effect was supported only for depressive symptoms. Specifically, self-compassion weakened the indirect association between materialism and depressive symptoms through BPNS, whereas the corresponding moderated mediation effect for life satisfaction was not statistically significant.

## 4. Discussion

This study explored the relationship between materialism and subjective well-being among Chinese higher vocational college students, as well as the mediating role of BPNS and the moderating role of self-compassion. In the present study, subjective well-being was operationalized using two complementary indicators: life satisfaction and depressive symptoms. Consistent with our hypotheses, materialism was significantly associated with lower life satisfaction and higher depressive symptoms, BPNS mediated the associations between materialism and both outcomes, and self-compassion moderated the association between BPNS and both life satisfaction and depressive symptoms. However, the moderated mediation effect was supported only for depressive symptoms, suggesting that self-compassion may be especially relevant in attenuating the indirect association between materialism and depressive symptoms through BPNS.

### 4.1. Materialism, BPNS, and Subjective Well-Being

The present study revealed that materialism was negatively associated with life satisfaction and positively associated with depressive symptoms, thereby corroborating findings from prior studies (Cleveland et al., 2023; Jiang et al., 2016; Mueller et al., 2011; Tsang et al., 2014). Although materialistic individuals often believe that happiness can be achieved through acquiring wealth and possessions (Richins & Dawson, 1992), the present findings suggest that such value orientations are associated with poorer well-being, as reflected in lower life satisfaction and higher depressive symptoms. This pattern may be particularly meaningful in the context of higher vocational education, where students are often preparing for employment and may face concerns about career prospects, social mobility, and future economic security (H. Li et al., 2024; X.-H. Wang et al., 2023; Zhai et al., 2021). In such a context, material success and social status may become especially salient standards for self-evaluation. These contextual features highlight the importance of examining the psychological mechanisms through which materialism is associated with subjective well-being in this population.

The results further showed that BPNS partially mediated the associations between materialism and both life satisfaction and depressive symptoms. Specifically, materialism was associated with lower levels of BPNS, which in turn was associated with reduced life satisfaction and increased depressive symptoms. This finding is consistent with SDT, which proposes that the satisfaction of the needs for autonomy, competence, and relatedness serves as essential psychological nutrients for well-being (Deci & Ryan, 2000). Materialistic individuals tend to prioritize external indicators of success, such as wealth, status, and appearance, often at the expense of intrinsically meaningful goals and relationships (Dittmar et al., 2014). Such an orientation may be linked to fewer experiences of autonomy, competence, and relatedness, which may in turn be associated with poorer subjective well-being. The present findings are also consistent with longitudinal evidence showing that psychological need satisfaction mediates the relationship between materialism and well-being (R. Wang et al., 2017). The fact that BPNS mediated the associations with both life satisfaction and depressive symptoms further suggests that psychological need satisfaction may be relevant to both cognitive and negative symptom-related indicators of subjective well-being. Nevertheless, the modest magnitude of the observed associations suggests that BPNS represents one possible mechanism linking materialism to subjective well-being, rather than a complete explanation of this relationship.

### 4.2. The Moderating Role of Self-Compassion

A central finding of the present study concerns the moderating role of self-compassion. The results showed that self-compassion moderated the associations between BPNS and both indicators of subjective well-being. However, the pattern of moderated mediation differed across the two outcomes.

For depressive symptoms, the BPNS × self-compassion interaction was significant, and the index of moderated mediation was also significant. The conditional indirect association from materialism to depressive symptoms through BPNS was significant at low and mean levels of self-compassion, but not at high levels of self-compassion. These findings suggest that self-compassion may attenuate the extent to which lower BPNS is linked to depressive symptoms within the materialism–BPNS–depressive symptoms pathway. This pattern is consistent with the view of self-compassion as a psychological resilience resource. BPNS reflects the extent to which individuals experience autonomy, competence, and relatedness in their daily lives (Chen et al., 2015; Ryan & Deci, 2000). When these needs are less satisfied, students may be more likely to experience psychological vulnerability, reduced adjustment, and emotional distress (Ryan & Deci, 2000; Vansteenkiste & Ryan, 2013). Self-compassion may help students respond to such difficulties with self-kindness, emotional balance, and recognition of common humanity, rather than with harsh self-criticism or over-identification with negative experiences (Neff, 2003a). In this way, self-compassion may weaken the association between lower BPNS and depressive symptoms. This interpretation is also consistent with previous research showing that self-compassion is closely related to lower self-criticism, reduced rumination, and better emotional adjustment (Barnard & Curry, 2011).

The findings for life satisfaction were more nuanced. Self-compassion significantly moderated the association between BPNS and life satisfaction, indicating that the positive association between BPNS and life satisfaction varied across levels of self-compassion. However, the index of moderated mediation was not statistically significant. Thus, although self-compassion was relevant to the BPNS–life satisfaction association, there was insufficient evidence that it significantly changed the indirect association from materialism to life satisfaction through BPNS. This distinction may reflect the different psychological nature of the two outcomes. Life satisfaction reflects a broad cognitive evaluation of one’s life (Diener et al., 1985; Diener et al., 1999), whereas depressive symptoms represent a negative symptom-related indicator of well-being that is closely connected to negative affective experiences, self-critical cognition, and psychological distress (Kroenke et al., 2001; McIntyre et al., 2018). Because self-compassion is particularly relevant to emotion regulation and adaptive responses to suffering, its protective role may be more evident for depressive symptoms than for general cognitive evaluations of life (Barnard & Curry, 2011; Neff, 2003a).

These findings provide a theoretical rationale for modeling self-compassion as a second-stage moderator. From a resilience perspective, self-compassion may be especially relevant to how students respond when their psychological needs are not fully satisfied. Thus, self-compassion may not primarily shape whether materialistic values are associated with lower BPNS, but may instead influence how strongly lower BPNS is associated with poorer well-being, particularly depressive symptoms. Supplementary analyses did not support the alternative first-stage moderation model in which self-compassion moderated the association between materialism and BPNS, further supporting the focus on the theoretically proposed second-stage moderation model.

### 4.3. Theoretical Contributions

The present study makes several theoretical contributions to the literature on materialism and well-being. First, the present study extends SDT-based research on materialism by providing context-specific evidence among Chinese higher vocational college students. Although previous studies have suggested that BPNS is an important mechanism linking materialism to well-being, less is known about whether this mechanism operates similarly among higher vocational college students, who may face distinctive concerns related to employability, social mobility, and future economic security (H. Li et al., 2024; X.-H. Wang et al., 2023; Zhai et al., 2021). By examining both life satisfaction and depressive symptoms, the present study shows that lower satisfaction of autonomy, competence, and relatedness may be relevant to both cognitive and negative symptom-related indicators of subjective well-being. In this sense, the present study contributes by contextualizing an established SDT-based mechanism within Chinese higher vocational education and by showing that this mechanism is relevant to both positive cognitive and negative symptom-related indicators of subjective well-being.

Second, the present study extends previous SDT-based research by integrating a resilience perspective into the materialism–well-being framework. Prior studies have mainly explained why materialism is associated with poorer well-being, whereas less attention has been given to psychological resources that may shape the strength of this association. The present findings showed that self-compassion moderated the associations between BPNS and both life satisfaction and depressive symptoms, suggesting that the well-being correlates of psychological need satisfaction may vary depending on students’ self-compassion. This finding indicates that students with different levels of self-compassion may not experience low BPNS in the same way. Students with higher self-compassion may respond to unmet psychological needs with greater self-kindness, emotional balance, and acceptance, whereas students with lower self-compassion may be more vulnerable to self-criticism, rumination, and emotional distress (Barnard & Curry, 2011; Neff, 2003a). Thus, the present study contributes to SDT by showing that personal psychological resources may influence how strongly BPNS is associated with well-being outcomes.

Third, the present study clarifies the different roles of self-compassion across life satisfaction and depressive symptoms. Although self-compassion moderated the associations between BPNS and both outcomes, the moderated mediation effect was supported only for depressive symptoms. Specifically, the indirect association between materialism and depressive symptoms through BPNS became weaker as self-compassion increased, whereas the corresponding moderated mediation effect for life satisfaction was not statistically significant. This pattern suggests that self-compassion may be especially relevant to negative symptom-related indicators of well-being (Barnard & Curry, 2011; Inwood & Ferrari, 2018; Neff, 2003a; Zessin et al., 2015). Life satisfaction reflects a broad cognitive evaluation of one’s life, whereas depressive symptoms involve negative affective and somatic symptoms (Kroenke et al., 2001). Because self-compassion is closely related to emotion regulation, reduced self-criticism, and adaptive responses to suffering, its protective role may be more evident in relation to depressive symptoms than in relation to general cognitive evaluations of life. Therefore, the present findings support the value of examining life satisfaction and depressive symptoms separately rather than treating them as interchangeable indicators of subjective well-being (Diener et al., 1999; Dittmar et al., 2014; Kroenke et al., 2001).

### 4.4. Practical Implications

The present findings have implications for mental health promotion and educational practice among higher vocational college students. Because the observed associations were generally modest, these implications should be interpreted as suggesting complementary rather than stand-alone intervention targets. The results suggest that promoting students’ subjective well-being may require attention not only to materialistic values but also to the psychological needs and emotional resources through which materialism is associated with well-being. In higher vocational colleges, this may be particularly important because students often face pressures related to employment preparation, future income, social mobility, and social comparison. These pressures may make material success especially salient, but the present findings suggest that supporting students’ autonomy, competence, relatedness, and self-compassion may help reduce the negative well-being correlates of such value orientations.

First, educators and mental health practitioners may create more need-supportive educational environments. Autonomy may be supported by providing meaningful choices, encouraging students to connect academic tasks with personal goals, and avoiding overly controlling feedback. Competence may be supported through clear expectations, constructive feedback, skill-building opportunities, and experiences of mastery, particularly in career-related and practical training contexts. Relatedness may be promoted through peer collaboration, mentoring programs, supportive teacher–student relationships, and inclusive campus activities. These practices may help higher vocational college students experience their academic and career preparation as more meaningful, effective, and socially connected.

Second, mental health programs may incorporate self-compassion-based components. Mindfulness-based interventions, self-compassion training, compassionate self-talk, self-compassionate letter writing, and reframing setbacks as common human experiences may help students respond more adaptively to academic pressure, employment concerns, interpersonal difficulties, and perceived failure. Given that the moderated mediation effect was supported only for depressive symptoms, self-compassion-based practices may be especially useful for students experiencing emotional distress or self-critical reactions to unmet psychological needs.

Finally, value clarification programs may help students reflect on the role of material goals in their lives. Rather than simply discouraging material aspirations, such programs can guide students to consider whether their goals support autonomy, competence, relatedness, and long-term well-being. This integrated approach may be more appropriate for higher vocational college students living in a consumer-oriented social context where material success and future economic security are highly salient.

### 4.5. Limitations and Future Directions

First, all variables were measured using self-report questionnaires, which may introduce social desirability bias and common method variance. Although Harman’s single-factor test and the single-factor CFA suggested that common method bias was unlikely to be a major concern, future studies should use multi-method and multi-informant designs where possible. For example, peer reports, behavioral indicators, or experience-sampling methods could provide more nuanced evidence regarding students’ daily need satisfaction, self-compassionate responses, and emotional states.

Second, the cross-sectional design prevents conclusions about temporal ordering or causality. Although the proposed model was theoretically grounded in SDT and resilience perspectives, alternative models are also plausible. For example, depressive symptoms may be associated with stronger materialistic values as a compensatory strategy, or lower life satisfaction may lead students to place greater emphasis on material success. Similarly, lower BPNS may both result from and contribute to materialistic orientations. Future studies should use longitudinal, cross-lagged, or experimental designs to clarify the directionality of these associations.

Third, the present study focused on higher vocational college students from southwestern China. Although this sample is theoretically meaningful given the rapid growth of consumer culture, academic pressure, and employment-related concerns in Chinese higher vocational education, the generalizability of the findings to students in research-oriented universities, other regions, other age groups, or different cultural contexts remains uncertain. Higher vocational college students may face distinctive developmental and contextual challenges, such as concerns about employability, social mobility, and future career prospects. Therefore, the present findings should be interpreted within this specific educational context. Future research should examine whether the same moderated mediation pattern emerges in more diverse samples, including students from comprehensive universities, other types of higher education institutions, and young adults outside educational settings.

Fourth, although the present study operationalized subjective well-being using life satisfaction and depressive symptoms, this approach does not capture all components of subjective well-being. In particular, the present study did not include direct measures of positive affect or general negative affect. Future studies should include more comprehensive measures of subjective well-being, such as life satisfaction, positive affect, negative affect, and additional indicators of psychological functioning, to further examine whether the present findings are robust across different components of well-being.

Fifth, although the use of a three-item life satisfaction measure was supported by additional validation evidence, future research may benefit from administering the full SWLS when possible. In addition, although the present study retained the theoretical dimensions of the main constructs, measurement issues related to reverse-worded items, cultural response styles, and method effects warrant further attention. Future studies should continue to examine the measurement structure of materialism and self-compassion scales in Chinese higher vocational college student samples and test whether these measures operate similarly across different cultural and educational contexts.

Finally, the present study conceptualized self-compassion as a moderator of the associations between BPNS and well-being outcomes, but other theoretical roles and model specifications are possible. Self-compassion may also function as a mediator, antecedent variable, covariate, or reciprocal process in the materialism–BPNS–well-being relationship. For example, higher self-compassion may support BPNS by helping students pursue more self-concordant goals and relationships, whereas depressive symptoms may also be associated with lower self-compassion through increased self-criticism and guilt. Future research should compare alternative models using longitudinal data. In addition, other psychological mechanisms, such as meaning in life, self-esteem, social comparison, and perceived social support, may further explain when and why materialism is associated with subjective well-being. In particular, meaning in life may be a promising direction for future research, as recent evidence suggests that it can serve as a mediator linking positive psychological resources with mental health outcomes (Adeeb et al., 2026). Integrating these factors with SDT and self-compassion frameworks may provide a more comprehensive understanding of higher vocational college students’ well-being.

## 5. Conclusions

In conclusion, the present study contributes to the growing literature on materialism and well-being by examining both a risk-related pathway and a resilience-related pathway among Chinese higher vocational college students. Materialism was associated with lower life satisfaction and higher depressive symptoms, and BPNS partially mediated these associations, suggesting that lower satisfaction of autonomy, competence, and relatedness may be one psychological mechanism linking materialistic values to poorer subjective well-being. By incorporating a resilience perspective into the Self-Determination Theory framework, the present study further showed that self-compassion moderated the associations between BPNS and both well-being indicators. However, the moderated mediation effect was supported only for depressive symptoms, indicating that self-compassion may be especially relevant in attenuating the indirect association between materialism and depressive symptoms through BPNS. Taken together, these findings provide a more comprehensive and context-specific understanding of materialism and subjective well-being among Chinese higher vocational college students and highlight potential targets for mental health promotion and educational practice, including need-supportive environments, self-compassion-based interventions, and value clarification programs. Given the cross-sectional nature of the data, however, the findings should be interpreted as theoretically informed associations rather than causal evidence. Future longitudinal and experimental studies are needed to further clarify the dynamic relationships among materialism, BPNS, self-compassion, life satisfaction, and depressive symptoms.

## Figures and Tables

**Table 1:** Descriptive Statistics and Correlations among Variables.

Variable	*M*	*SD*	1	2	3	4	5	6	7	8	9	10
1. Materialism	2.92	0.50	1.00									
2. BPNS	3.64	0.62	−0.20 **	1.00								
3. Life satisfaction	3.26	0.81	−0.26 **	0.44 **	1.00							
4. Depressive symptoms	0.41	0.44	0.23 **	−0.45 **	−0.39 **	1.00						
5. Self-compassion	3.37	0.57	−0.33 **	0.48 **	0.40 **	−0.45 **	1.00					
6. Age	18.79	0.96	−0.06 **	0.06 **	0.00	−0.05 **	0.05 **	1.00				
7. Gender	0.63	0.48	0.21 **	−0.01	0.00	−0.03	−0.05 **	−0.04 *	1.00			
8. Subjective SES	1.22	0.78	0.01	0.11 **	0.36 **	−0.12 **	0.09 **	−0.07 **	0.02	1.00		
9. Residence	0.67	0.47	−0.07 **	−0.06 **	−0.13 **	0.02	−0.04 **	0.05 **	0.03	−0.24 **	1.00	
10. Only-child status	0.22	0.41	−0.02	0.07 **	0.10 **	−0.01	0.04 *	0.02	−0.09 **	0.12 **	−0.25 **	1.00

Note. *N* = 4012. BPNS = basic psychological need satisfaction; subjective SES = subjective socioeconomic status. Gender was coded as 0 = male and 1 = female. Residence was coded as 0 = urban and 1 = rural. Only-child status was coded as 0 = non-only child and 1 = only child. Subjective SES was coded as 0 = facing difficulties, 1 = below average, 2 = average, 3 = very comfortable. * *p* < 0.05, ** *p* < 0.01.

## Data Availability

The data that support the findings of this study are available on reasonable request from the corresponding author. The data are not publicly available due to privacy or ethical restrictions.

## Abbreviations

The following abbreviations are used in this manuscript:

SDT	Self-Determination Theory
BPNS	Basic Psychological Need Satisfaction
SES	Socioeconomic Status
